# Live bacterial vaccines – a review and identification of potential hazards

**DOI:** 10.1186/1475-2859-5-23

**Published:** 2006-06-23

**Authors:** Ann Detmer, Jacob Glenting

**Affiliations:** 1 Danish Toxicology Centre, Hørsholm, Denmark; 2 Bioneer A/S, Hørsholm, Denmark

## Abstract

The use of live bacteria to induce an immune response to itself or to a carried vaccine component is an attractive vaccine strategy. Advantages of live bacterial vaccines include their mimicry of a natural infection, intrinsic adjuvant properties and their possibility to be administered orally. Derivatives of pathogenic and non-pathogenic food related bacteria are currently being evaluated as live vaccines. However, pathogenic bacteria demands for attenuation to weaken its virulence. The use of bacteria as vaccine delivery vehicles implies construction of recombinant strains that contain the gene cassette encoding the antigen. With the increased knowledge of mucosal immunity and the availability of genetic tools for heterologous gene expression the concept of live vaccine vehicles gains renewed interest. However, administration of live bacterial vaccines poses some risks. In addition, vaccination using recombinant bacteria results in the release of live recombinant organisms into nature. This places these vaccines in the debate on application of genetically modified organisms. In this review we give an overview of live bacterial vaccines on the market and describe the development of new live vaccines with a focus on attenuated bacteria and food-related lactic acid bacteria. Furthermore, we outline the safety concerns and identify the hazards associated with live bacterial vaccines and try to give some suggestions of what to consider during their development.

## Background

Live vaccines have played a critical role from the beginning of vaccinology. Indeed, the very first vaccination experiment in the Western world was Jenner's inoculation of a boy with the milder cowpox virus to protect against the deadly smallpox. Although effective the technology has safety problems associated with the risk of reversion to a virulent organism and the possibility of causing disease in immune compromised individuals. Within the last 20 years the concept of live vaccines gains renewed interest due to our increased immunological understanding and the availability of molecular techniques making the construction of safer live vaccines possible. This opens for the development of new live bacterial vaccines that can avoid the downsides of parenterally administered vaccine because it (i) mimics the route of entry of many pathogens and stimulate the mucosal immune response (ii) can be administered orally or nasally avoiding the risk associated with contaminated needles and need for a professional healthcare infra structure (iii) has a simple down stream processing. Broadly, live bacterial vaccines can be classified as a self-limiting asymptomatic organism stimulating an immune response to one or more expressed antigens.

Furthermore, live bacterial vaccines can be designed to induce an immune response to itself or to a carried heterologous antigen. A non-virulent or attenuated derivative of the pathogen is used to induce a response to the bacterium itself. When used as a vaccine vehicle the bacterium expresses an antigen from another species. Most commonly, these vaccine vehicles are based on either attenuated pathogens or bacteria used in the food industry. Both classes of bacteria deliver the vaccine component to the immune system whereby immunization may benefit from the bacterium's intrinsic adjuvant. The vaccine component to be delivered can be either protein or DNA. In addition, the vaccine component may be a classical antigen but may also be allergens or therapeutics. A recent development is the use of invasive bacteria for the delivery of plasmid DNA vaccines to mammalian cells obtaining *in vivo *synthesis of the plasmid-encoded antigen. As such, the applications of live bacterial vaccines are extensive and has lead to more than 2000 published papers. However, only very few of the promising candidates have survived the licensing process and become registered [1] illuminating the difficulty in developing a commercial live vaccine. One typhoid vaccine (Ty21a) contains live attenuated *Salmonella typhi *and is administered orally either as a liquid or as acid resistant capsules. Both formulations require three doses within one week to give immunity. The other registered vaccine based on live bacteria is against cholera and is given orally as a single dose of attenuated *Vibrio cholerae *(CVD 103-HgR) in liquid formulation. This vaccine is used in a lower dose (5 × 10^8 ^live bacteria) for travellers from non-endemic regions and a one log higher dose for residents in endemic regions (5 × 10^9 ^live bacteria). The very few examples of live bacterial vaccines on the market may be due to lack of success in clinical trials. However, we believe that the safety of these vaccines is another issue. Indeed, prophylactic vaccines are given to healthy people and despite excellent safety record they remain targets of un-substantiated allegations by anti vaccine movements. Furthermore, future live vaccines will most likely be either targeted mutagenised or equipped with foreign antigens and therefore considered recombinant. As such, they fall into the debate on releasing genetically modified organisms into nature. The feasibility of this new vaccine strategy will therefore in particular depend on considerations of safety issues. We believe that considering safety issues alongside the scientific consideration early in vaccine development will facilitate its public acceptance and its entrance to the market. We therefore felt compelled to outline a review about live vaccines and their safety aspects.

### Attenuated pathogens as vaccines and vaccine vehicles

Lindberg [2] has excellently reviewed the history of live bacterial vaccines. The first use of a live bacterial vaccine was in Spain in 1884 and consisted of a subcutaneous injection of weakened *Vibrio cholerae*. This study was followed a few years later by field trials in India with a more efficacious *V. cholerae *vaccine, however still parenteral. The first live oral *V. cholerae *vaccine candidate did not appear until the 1980s. Later the *V. cholerae *strain CVD 103 Hg-R has been found to be both safe and immunogenic after a single oral dose. In 1996 a bivalent vaccine waspresented including two strains of *V. cholerae *called CVD 103 Hg-R and CVD 111 [3]. However, later on problems with attenuation of strain CVD 111 appeared [4]. The development of the other registered live bacterial vaccine began Hg-in the early 1970s using various live attenuated *S. typhi *to vaccinate against typhoid fever. One proposed strain was made streptomycin-dependent, but failed to be efficacious in freeze-dried formulation [5]. Furthermore, the strain was genetically unstable and reverted to virulence. Another *S. typhi *strain (Ty21a) with a defect *galE *gene, as well as other not defined mutations, requires an external source of galactose. This strain was extensively evaluated in several field trials and has shown excellent safety record [6]. Later, other auxotrophic strains unable to synthesise essential compounds like aromatic amino acids were developed and tested on human volunteers with variable safety and immunogenicity results [7-10]. Attenuated live vaccines to prevent shigellosis have also been proposed. Both genetically engineered or selected mutants of *Shigella *have been tried but showed side effects in clinical trials and points to the need of additional attenuation without hampering immunogenicity [11-13]. Kotloff et al attenuated the guanine auxotrophic *Shigella flexneri *2a further by deleting two genes encoding enterotoxins [14]. In a phase 1 trial this strain with inactivated enterotoxin genes was better tolerated but still immunogenic compared to the guanine auxotrophic strain that contain active entoroxins.

Recombinant *Shigella *has also been proposed as a vaccine vehicle [15]. Pathogenic *Shigella *has a virulence plasmid encoding proteins involved in thesecretion apparatus and proteins necessary for the entry process into human cells. This invasive capacity can be used to deliver plasmid DNA vaccines into mammalian cells [16]. Here, the delivered plasmid DNA encodes an antigen, which is expressed by the protein synthesis apparatus of the infected cells. Diaminopimelate *Shigella *auxotrophs undergo lysis unless diaminopimelate is present in the growth media [16]. Human cells contain low amounts of diaminopimelate and upon entry the *Shigella *mutant lyse making the delivery of vaccine components more effective. Other attenuated bacteria have also been tested as vaccine vehicles of various proteins and plasmid DNA (Table 1). In conclusion, the mimicry of natural infection makes attenuated bacteria effective. The ability to deliver vaccine components of different origins like e.g., HIV [15,17,18] or piece of parasitic DNA [19] or gamete specific antigen [20] make attenuated bacteria a versatile vaccinology tool. However, in spite of the efforts in constructing attenuated pathogens for use as bacterial vaccine vehicles none of them has reached the market yet.

**Table 1:** Attenuated bacteria as vaccine vehicles

Vaccine strain	Attenuation	Foreign insert	Model	Ref.
*Shigella flexneri*	Δ*asd*	pCMVβ	Guinea pig, *in vitro*	[80]
	Δ*asd*	CS3 and LTB/STm	Mouse	[81]
	Δ*rfb*F	HIV-1 SF2Gag	Mouse	[17]
	Δ*dap*A Δ*dap*B	β-gal, gene vaccine	*In vitro*	[16]
	Δ*aro*A Δ*isc*A	gp120, gene vaccine	Mouse	[15]

*Salmonella enterica*	Δ*aro*A	pCMVβ, pCMV*act*A and pCMV*hly*	*In vitro*, mouse	[82]
	Δ*aro*A Δ*aro*D	*C. tetani *TTFC	Mouse	[83]
	Δ*aro*A Δ*htr*A	TTFC	Mouse	[83]
	Δ*aro*A+others	GFP+cytokines	Mouse	[84]
	Δ*cya *Δ*crp *Δ*asd*	SP10 cDNA	Mouse	[20]
	*Gal*E + unspecified	*H. pylori*, *ure*AB	Human	[85]

*Yersinia enterocolitica*	pYV^-^	*B. abortus*, P39	Mouse	[86]
	pYV^-^	Ova	Mouse	[87]

*Listeria monocytogenes*	Δ*act*A	*Leichmania major*	Mouse	[88]
	Δ*act*A	LCM virus	Mouse	[89]
	Δ*dal *Δ*dat*	HIV-1 gag gene vaccine	Mouse	[90]
	Δ*2*	*M. bovis *gene vaccine	Mouse	[91]

*Bordetella bronhiseptica*	Δ*aro*A	TTFC	Mouse	[92]

*Erysipelotrix rhusiopatie*	Tn916^-^	*M. hyopneumonie*	Mouse, pig	[93]

*Mycobacterium bovis*	unspecified	*P. falciparum*, CSP	Mouse	[94]

*Brucella abortus*	Rough mutant (O^-^)	*lac*Z or HSP65	Mouse	[95]

### Lactic acid bacteria as vaccine vehicles

The potential of using lactic acid bacteria (LAB) for the delivery of vaccine components is less exploited than attenuated pathogens. Due to their safe status and the availability of genetic tools for recombinant gene expression LAB are attractive for use as vaccine vehicles. Furthermore, their non-pathogenic status circumvents the need to construct attenuated mutants. However, LAB are non-invasive and the vaccine delivery to antigen presenting cells may be less effective than invasive bacteria. Still, antigen specific immune responses have been obtained with several LAB (Table 2). Geoffroy et al [21] used a green fluorescent protein to visualize the phagocytosis of *Lactobacillus plantarum *by macrophages *in vitro *and in mice. Macrophages act as antigen presenting cells and this can explain a possible way to at least elicit a ClassII MHC receptor presentation of the antigen. Even though the transit time of *Lactococcus lactis *through the intestine is less than 24 h in mice [22], a potent immune response has been obtained with several antigens including tetanus toxin fragment C (TTFC). Surprisingly, a similar response was induced using dead or alive *Lactococcus *suggesting that *in situ *antigen synthesis is not essential [23]. A slightly better result was in the same study obtained with *L. plantarum*, but also here a similar response was induced from living or UV-light inactivated cells.

**Table 2:** LAB as vaccine vehicles

Vaccine strain	Foreign insert	Model	Ref.
*Lactococcus lactis*	*C. tetani *TTFC	Mouse	[23,96]
	TTFC+IL-2 or IL-6	Mouse	[97]
	Human IL-10	Mouse	[39]
	*H. pylori ure*B	Mouse	[98]
	*B. abortu*s L7/L12	Mouse	[99]
	*S. pneumonie *CPS	Mouse	[100]
	Rotavirus vp7	Mouse	[101]
	B-lactoglobulin	Mouse	[102]
	HIV-1 gp120	Mouse	[103]
	Malaria MSP-1	Mouse	[104]
	SARS Nucleocapsid protein	*In vitro*	[105]
	*E. rhusiopathiae *SpaA	Mouse	[106]

*Lactobacillus plantarum*	TTFC	Mouse	[107]
	Allergen Der p1	Mouse	[36]
	*H. pylori *(*ureB*)	Mouse	[108]

*Streptococcus gordonii*	Antibody	Rat	[34]
	Hornet venom Ag5.2	Mouse	[109]
	TTFC	Mouse	[110]

*Lactobacillus casei*	*B. anthracis *(protective Ag)	*In vitro*	[111]
	SARS spike protein	Mouse	[112]
	Human papillomavirus L1	*In vitro*	[113]
	Coronavirus S glycoprotein	Mouse	[114]
	*S. pneumonie *PsaA PspA	*In vitro*	[115]

*Lactobacillus zeae*	Antibody	Rat	[33]

*Lactobacillus johnsonii*	TTFC mimotope	Mouse	[116]

### Active vaccination using LAB

The prospect of using live LAB as vaccine carriers has been reviewed [24,25]. The most frequently used model antigen is TTFC in which good results have been obtained both in intranasal and oral mice models using strains of *L. plantarum *and *L. lactis *[23,26]. Grangette et al [27] tested cytoplasmic expression of TTFC antigen in both *L. plantarum *and *L. lactis *showing protective effect in an oral mouse model. Shaw et al [28] tested both cytoplasmic and surface associated expression of same TTFC antigen and found that cytoplasmic expression was superior to surface exposed TTFC in *L. lactis*. In contrast, Bermúdez-Humarán et al [29] tested human papillomavirus type 16 E7 antigen sorted in different cellular compartments and found cell wall-anchored antigen to induce the most potent immune response. The different outcome of these experiments may be explained by different stability of surface exposed TTFC and E7 antigen. Intracellular expression of a labile antigen can protect it from proteolytic degradation and environmental stress encountered at the mucosal surfaces. Genetic modification of the LAB cell wall rendering the strain more permeable increases the *in vivo *release of cytoplasmic TTFC antigen and was tested by Grangette et al [27]. When administered orally these alanin racemase mutants were more immunogenic than their wild type counterparts. One explanation could be that oral immunization is very dependant on a sufficiently large dose of the antigen [27].

The use of live LAB as carriers of DNA vaccines has until now not been an option as they are non-invasive and therefore inefficiently deliver the plasmid DNA to the cytoplasma of antigen presenting cells. Recently Guimarães et al [30] developed *L. lactis *expressing cell wall-anchored internalin from *Listeria monocytogenes*. This *L. lactis inlA*+ strain has been shown to enter eukaryotic cells *in vitro*, but also *in vivo *using an oral guinea pig model. To determine the tropism of recombinant invasive strains Critchley-Thorne el al used a perfusion bath with murine ileal tissue and tested an invasive *E. coli *vaccine candidate [31]. Although change of tropism of a bacterial carrier opens for targeted delivery it introduces new safety issues that should be addressed by persistence and distribution studies of the bacterial strain after vaccination.

Active vaccination using recombinant *L. johnsonii *to treat allergy has been suggested [32]. IgE epitopes was fused to proteinase PrtB and cell wall-anchored. Subcutaneous and intranasal immunization of mice induced a systemic IgG response against human IgE. As such, allergy-inducing IgE may be cleared by IgG antibodies induced by the recombinant *L. johnsonii*. However, it remains to be proven if these antibodies are protective in human patients.

In conclusion, LAB has been successfully used for active vaccination of animals like rodents (Table 2). Whether LAB will be effective as a mucosal vaccine in humans can only be answered by clinical trials. Furthermore, as the dose of recombinant LAB needed to elicit immune responses in animals is high it is unknown if the necessary dose for use in humans will be feasible and cost effective.

### Passive immunization using LAB

Protection by preformed antibodies or antibody fragments is called passive vaccination. The pioneer experiments were based on injection of antisera produced by immunized animals like horse or sheep to combat for example rattlesnake venom. Recently, passive immunity was delivered using lactobacilli that secretes single-chain antibodies [33]. In a rat caries model, colonisation of the mouth with a *L. zeae *expressing a single-chain antibody fragment recognizing the adhesion molecule of *Streptococcus mutans *decreased the number of *S. mutans *and reduced the development of caries. Recombinant *Streptococcus gordonii *displaying a microbiocidal single-chain antibody (H6) has been used to treat vaginal candidiasis in a rat model [34]. Although passive immunity has limits in its temporary nature, these results suggest that LAB elegantly can be used for the delivery of neutralising antibodies at mucosal sites.

### Allergy vaccines using LAB expressing allergens

For a normal vaccination against an infectious disease, induction of tolerance to the infectious agent is considered a side effect. This side effect is more prone to happen when vaccinating early in life [35]. However, induction of tolerance can have positive clinical implications when the purpose is to treat allergy. In a mouse model the use of a recombinant *L. plantarum *expressing the house dust mite allergen Der p1 as a fusion protein in the cytoplasm inhibited house dust mite-specific T-cell responses [36]. In this study mice were sensitized by immunization with the house dust mite peptide and then given either *L. plantarum *expressing Der p1 or *L. plantarum *without Der p1. Both strains inhibited IFN-γ production by T cells. But the decrease in production of-5 was only seen for the *L. plantarum *expressing the Der p1 peptide antigen. This indicates that the lactobacilli strain expressing Der p1 can suppress the cytokine milieu promoting the Th2 allergic response. Another example of the strain specific effect of LAB on induction and maintenance of oral tolerance has been shown using ί-lactoglobulin and gnotobiotic mice [37]. In this study *L. paracasei *(NCC 2461) was more effective to induce and maintain oral tolerance in gnotobiotic mice than was *L. johnsonii *(NCC 533). The allergen can also be co-administered instead of recombinant expressed by the LAB. Mucosal co-application of *L. plantarum *or *L. lactis *together with birch pollen allergen Bet v1 shifted the immune response towards an anti-allergic Th1 response both in sensitized and un-sensitized animals [38]. Recombinant strains expressing immune polarizing cytokines like IL-10 have also been developed and *in vivo *effects in both mice [39] and pigs [40] have been observed. More knowledge on the mechanisms behind skewing the immune response is however needed to select the proper strain with anti allergic immune polarization. Furthermore, the immune regulatory effect of one strain of LAB may differ in allergic and non-allergic individuals. A down regulation in allergic persons and an immune stimulating effect in normal persons was observed when using same strain of LAB [41].

### Immune stimulatory effects of LAB

Among LAB's effect on the immune system there is a strain dependent induction of cytokines. Different LAB strains induce distinct mucosal cytokine profiles in BALB/c mice [42] pointing at the importance of using one strain for immune induction and another for induction of tolerance or a partial down regulation of the immune system. The same authors [43] also indicate growth phase dependent differences of orally administered LAB strains on the IgG1 (Th2)/IgG2a (Th1) antibody ratio in mice further complicating the process of choosing the proper strain for specific modulation of the immune response. Adding to the complexity of these observations, a human study has shown that non-specific immune modulation by a given strain of *L. rhamnosus *(GG, ATCC 53103) differs in healthy and allergic subjects. In healthy persons the strain was immune stimulatory whereas in allergic persons it down-regulated an inflammatory response [44]. Interactions between different LAB strains can also interfere with the *in vitro *production of cytokines by dendritic cells [45]. As is shown in another study [46], two different lactobacilli with similar probiotic properties *in vitro *were shown to elicit divergent patterns of colonisation and immune response in germfree mice. Further evidence for an immune modulating effect is seen when either *L. lactis *or *L. plantarum *was used in a mouse model of birch pollen allergy [38]. In combination with birch pollen allergen Bet v1 both strains skewed the immune response from Th2 to Th1 in sensitised mice as indicated by the IFN-γ/IL-5 ratio. The immune polarizing effect of LAB has also been observed in humans. A clinical trial showed a strain dependent immune modulation of two different LAB strains when administered together with an oral *S. typhi *vaccine (Ty21a) [47]. Here, thirty healthy volunteers were randomised into three groups receiving *L. rhamnosus *GG, *L. lactis *or placebo for 7 days. On days 1, 3 and 5 the Ty21a vaccine was given orally. Analysis showed a higher number of specific IgA-secreting cells in the group receiving *L. rhamnosus *GG and a higher CR3 receptor expression on neutrophils in the group receiving *L. lactis*. A partial down regulation of the immune system has also been observed. Atopic children receiving 2 × 10^10 ^*L. rhamnosus *GG daily for 30 days enhanced their IL-10 production in sera as well as in mitogen-induced peripheral blood mononuclear cells [41].

It can be concluded that immune polarization towards either a Th2 or a Th1 response can be obtained using different LAB. As such the intrinsic immune modulatory capacity of the LAB must be evaluated and selected to fit the purpose of vaccination.

### Safety concerns of the bacterial vaccine strain

Several safety concerns of the bacterial vaccine strain have been raised (Table 3). Before using pathogenic bacteria for vaccination purposes, its pathogenicity must be weakened via attenuation. Attenuation usually involves deletion of essential virulence factors or mutation of genes encoding metabolic enzymes whose function is essential for survival outside the laboratory. Inactivation of a metabolic gene has the advantage that the bacteria still express virulence determinants important to elicit a protective immune response. Appropriate stable auxotrophic strains are usually not able to replicate in the human body and can safely be used even in immune compromised individuals. Defined deletions of at least two metabolic essential genes are usually used [2] and decrease the probability of reversion to virulence. To reduce the risk of spreading foreign genetic material to the environment the antigen encoding gene cassette can be inserted into the chromosome replacing the metabolic essential gene. If the bacterium acquires the deleted gene it will automatically loose the antigen-encoding cassette. The use of antibiotic resistance genes as marker genes in vaccines is not encouraged as these genes can transfer to in the end humans and thus hamper the use of therapeutic antibiotics. Different alternatives to antibiotic resistance marker genes have been published and should be used as soon as possible in the developmental process of a vaccine [48-50].

**Table 3:** Safety concerns of the vaccine strain

Systemic disturbance	Systemic infection
	Conversion from avirulent to virulent bacterium
	Translocation to organs
	Disturbance of digestive processes
	Inhibition of bacterial production of nutrients
Immune system	Absorption of allergens through the intestinal epithelium
	Induction of tolerance to pathogen instead of immunity
	Induction or potentiation of autoimmunity
	Bacterial mimicry of self-antigen

Metabolites	Production of harmful/undesired metabolites including enzymatic activities
	Breakdown of chemicals to toxic metabolites

Implications for natural flora in GI tract	Permanent colonisation of cell substrate in the intestine
	Gene/plasmid transfer to host's indigenous flora
	"Competitive exclusion" of indigenous flora

Unintentional transferral of cell substrate	Unintentional transfer to other individuals
	Unintentional transfer to and viability/propagation in environments other than the intestines

Contamination	Extraneous or perceived adventitious DNA components should be removed (possibility of oncogenicity).

Another concern using live bacterial vaccines is the onset of autoimmune responses like arthritis especially in patients with the HLA-B27 tissue type [51]. However, the risk is certainly lower than after natural infection. The occurrence of such side effects can best be followed by post launch monitoring and must always be evaluated against the health risks associated to the disease itself. A theoretical side effect of vaccines is the possible induction of autoimmune reactions. However, there is no recommendation to avoid vaccination of people with an ongoing autoimmune disease like rheumatoid arthritis or systemic lupus erythematosus if vaccination otherwise is motivated [52]. In contrast, immune-compromised hosts can have difficulties in handling replicating live attenuated vaccines and should therefore not be vaccinated with such vaccines. However, new ways of further attenuating bacteria like combining auxotrophy with deletions of virulence genes [14] may open for the use of live vaccines to immune-compromised hosts. In addition, immune-compromised people close to hosts vaccinated with live attenuated vaccines should be aware of the risk of cross contamination with the vaccine strain.

Adverse examples of human live vaccine strains causing death and illness among domesticated animals are rare but existent. In Mongolia in autumn 1979 the H1N1 influenza A vaccine virus may have caused a severe influenza epizootic among camels [53]. No examples of human bacterial vaccines causing problems among animals have been found in the literature but the possibility exists and has to be both tested and evaluated before release of a live bacterial vaccine. In general the spread of live bacterial vaccines to the environment is a concern. However, attenuated human pathogens are usually not adapted to live outside its host. Therefore survival in the environment is usually short. Vaccines based on recombinantLAB may result in the release of these bacteria in nature, as LAB are more suited to survive in the nature. Also here the use of auxotrophic mutants unable to replicate in the environment may be the answer. Releasing gene-modified organisms into the environment can cause debate and precautions to eliminate its spread are essential. To avoid escape into the environment of the genetically modified organism, Steidler et al. [40] replaced the *thyA *gene with the expressioncassette for human IL-10. As a consequence, the *L. lactis *mutant is dependant on thymidine or thymine for growth, which is present in low amounts in nature and in the human body. Furthermore, acquirement of an intact *thyA *gene would recombine the transgene out of the genome, resulting in reversion to its wild type state.

### Safety concerns of the antigen encoding sequence

In live bacterial vaccines the antigen-encoding gene is either plasmid located or integrated in to the chromosome. In both cases several safety concerns can be raised (Table 4). For plasmid-encoded antigens the fate of the plasmid in the vaccinee must be evaluated. The use of a prokaryote plasmid replication unit of narrow host range can limit the horizontal plasmid transfer to other bacteria present in the vaccinated individual and prevent undesired persistence of the plasmid. In particular for plasmid DNA vaccines a study should identify which cells take up and/or express the DNA and what is the fate of the DNA within those cells as well as for how long the DNA persists in the cells [54]. Nasal administration of a naked DNA-vaccine in mice led to some accumulation of plasmid DNA in the brain [55] illustrating the diffusion of the plasmid after immunization. The amount of accumulating plasmid that is acceptable outside the target cells needs to be further clarified.

**Table 4:** Safety concerns of the antigen encoding sequence

For protein and DNA vaccines	Transfer of undesired genes via plasmid
	Transfer of vector to indigenous flora
	Open reading frames coding for injurious peptides (allergens)
	Imprecise transcription and translation
Specifically for DNA-vaccines	Persistence of DNA
	Permanent expression of the foreign antigen
	Formation of anti-DNA antibodies
	Transformation event
	Spread of antibiotic resistance genes

The recombinant plasmid harboured by bacterial vaccine vehicles may integrate in the genome of the recipient and potentially cause oncogenesesis. Concerns about the potential oncogenicity of biological products like continuous cell line products (CCL), DNA vaccines and gene therapy products have been raised [54]. In CCLs foreign DNA should be avoided in the final product and a limit has been defined as for maximal residual amount per human dose. In DNA vaccines DNA is obvious present but insertion of DNA should be avoided. Finally in the gene therapy product DNA is both present and inserted but insertional oncogenesis should be avoided. Integration of foreign DNA into the host genome is by definition insertional mutagenesis and can induce oncogenesis. There are three ways the extraneous DNA can lead to transformation [54]: insertion of an active oncogene, insertional activation of a host proto-oncogene, and by insertional deactivation of a host suppressor gene. The mechanism behind DNA integration into the chromosome is either by random integration, homologous recombination or retroviral insertion [56]. The most probable cause of unwanted integration is by random integration which occurs at a frequency of approximately 10^-4 ^[54]. Unwanted integration by homologous recombination and retroviral insertion can be avoided by omission of sequences necessary for insertion [57]. Analysing the antigen encoding unit carried by the bacteria for human homologous sequences and eliminating these can limit the integrative possibility. Although not similar to vaccination with bacteria the clinical trials using retroviral therapy can give some indications of the hazards of DNA integration [58]. Indeed, activation of oncogenes is a risk associated with retroviral vaccination [59]. The report of adverse effects in a French gene therapy study, where 2 out of 10 patients developed leukaemia within 3 years of [60,61], illustrates occurrence of such a transformation event by activation of a proto-oncogene. Calculation of the probability of a harmful effect due to integration of foreign DNA into host genome has been performed and was found to be less than 10^-16 ^to 10^-19 ^per DNA molecule [62]. This frequency must be put in relation to the spontaneous mutation frequency which has been estimated in humans to occur at the rate of 1 in every 50 million nucleotides incorporated during DNA replication. This means that a human cell with 6 × 10^9 ^base pairs will contain 120 new mutations [63].

Possible insertions into the chromosome can be tested by PCR techniques [64,65]. However, insertion due to random integration can be difficult to detect this way [64]. Furthermore, insertion of foreign DNA can effect gene activity at sites remote from insertion [66]. Different animal trial has foreseen possible adverse effects like in the following two examples. Foreign DNA ingested by mice has been shown to be covalently linked to mouse DNA [67]. Foreign DNA has also been shown in association with chromosomes in fetuses born by mice fed orally with bacteriophage M13 DNA [68]. There is however, no evidence for a germ line transmission of ingested foreign DNA [66]. The *de novo *methylation that frequently occurs with integrated foreign DNA has been suggested as being a natural defence mechanism [69].

In conclusion, integration of the plasmid harboured by bacterial vaccine vehicles is a potential hazard. Integration of gene therapy vectors has been observed, but omitting sequences driving the insertion may limit the possibility for integration of the plasmid carried by the bacterium. Plasmids for heterologous gene expression are usually preferred due to its multi copy nature and higher gene dosage. However, placing the antigen encoding genes on to the bacterial chromosome may limit the spread of the genes. The route of administration of the vaccine may also be important when evaluating hazards. As live bacterial vaccines is fit for mucosal administration one must remember that ingestion of foreign DNA does occur every day with our food and is as such not new.

Peptides can be absorbed through the mucosa and some may induce an allergic reaction. The existence of genes in the bacterial vaccine coding for such potential allergens or other injurious peptides can be checked beforehand searching for homologies to known allergens, as the full sequence of the bacteria and plasmid should be known.

Vaccination using live bacterial vaccines or exposure to the natural infections can lead to the formation of auto reactive antibodies, especially in people prone to autoimmune diseases. However, the half life of the induced auto antibodies is usually short [70] and their specificity usually polyclonal [71]. Several authors have tried to elucidate the possibility of a link between autoimmunity and vaccination [70,72-77] and much controversy in this matter is still existing. However, convincing data establishing a link between vaccination and autoimmunity in man are still not presented. In a mouse model a difference in clinical outcome was observed in two different mouse strains in relation with auto-antibodies induced by vaccination with dendritic cells loaded with apoptotic thymocytes [78]. In normal BALB/c mice the presence of post vaccination autoantibodies was not associated with any clinical or histological sign of autoimmunity. However, in mice prone to autoimmunity (NZBxNZW) F_1 _a severe pathology attributed to autoimmunity was observed. This difference in outcome attributed to the difference in genotype has also been observed in humans and it can be concluded that susceptibility to autoimmunity is determined more by genetic factors than by vaccine challenge despite the formation of post vaccination auto-antibodies [77]. A vaccination or treatment with adjuvant can also activate regulatory T cells and can thus be used as a method to prevent autoimmune disease if applied at the right time [79]. In the future tailor-made vaccines might be the solution for individuals with a genetic profile prone to autoimmunity.

## Conclusion

Both attenuated bacteria like salmonella and food related lactic acid bacteria have been developed as live vaccines suitable for oral administration. Today, live vaccines based on attenuated *S. typhi *and *V. cholerae *are available. The development of bacterial vaccine vehicles carrying a heterologous gene or a DNA vaccine is more problematic and none has yet reached the market. Several bacteria have been suggested as vaccine vehicles and especially lactic acid bacteria are promising. Their safe status and immune modulating capacity have been tested using diverse vaccine components like antigens from infectious diseases, allergy promoting proteins and therapeutic antibodies. However, considerable safety issues against live vaccine vehicles can be raised. Their recombinant nature calls for a bio containment strategy and auxotroph mutants may be the answer. The bacterial host must be fully sequenced and evaluated using bioinformatics tools for the production of allergy inducing peptides. The antigen encoding gene cassette must be sequenced and homologies to self proteins or allergy inducing proteins should be addressed. Especially bacteria carrying recombinant plasmids the probability of horizontal gene transfer to other bacteria present should be avoided by using host restricted replication units. Furthermore, the plasmids should be evaluated for sequences facilitating integration into the human genome.

## Authors' contributions

The authors contributed equally to this work.
